# 4-Carbamoylpiperidinium 5-nitro­salicylate

**DOI:** 10.1107/S1600536810050129

**Published:** 2010-12-15

**Authors:** Graham Smith, Urs D. Wermuth

**Affiliations:** a Faculty of Science and Technology, Queensland University of Technology, GPO Box 2434, Brisbane, Queensland 4001, Australia

## Abstract

In the crystal structure of the title compound, C_6_H_13_N_2_O^+^·C_7_H_4_NO_5_^−^, the isonipecotamide cations and the 5-nitro­salicylate anions form hydrogen-bonded chain substructures through head-to-tail piperidinium–carboxyl­ate N—H⋯O hydrogen bonds and through centrosymmetric cyclic head-to-head amide–amide hydrogen-bonding associations [graph set *R*_2_^2^(8)]. These chains are cross-linked by amide–carboxyl­ate N—H⋯O and piperidinium–nitro N—H⋯O associations, giving a sheet structure.

## Related literature

For structural data on isonipecotamide salts, see: Smith *et al.* (2010 ▶); Smith & Wermuth (2010*a* ▶,*b* ▶,*c* ▶,*d* ▶). For structures of 5-nitro­salicylates, see: Smith *et al.* (2005 ▶). For hydrogen-bonding graph-set and motif classification, see: Etter *et al.* (1990 ▶); Allen *et al.* (1998 ▶).
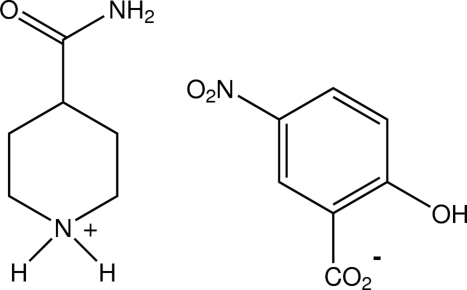


## Experimental

### 

#### Crystal data

C_6_H_13_N_2_O^+^·C_7_H_4_NO_5_^−^
*M* *_r_* = 311.30
Monoclinic, 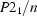

*a* = 15.0442 (10) Å
*b* = 5.5851 (3) Å
*c* = 17.1939 (10) Å
β = 91.466 (6)°
*V* = 1444.22 (15) Å^3^
*Z* = 4
Mo *K*α radiation
μ = 0.12 mm^−1^
*T* = 200 K
0.40 × 0.25 × 0.16 mm

#### Data collection

Oxford Diffraction Gemini-S CCD-detector diffractometer
Absorption correction: multi-scan (*CrysAlis PRO*; Oxford Diffraction, 2010 ▶) *T*_min_ = 0.912, *T*_max_ = 0.980
9191 measured reflections
2833 independent reflections
1850 reflections with *I* > 2σ(*I*)
*R*_int_ = 0.031

#### Refinement

*R*[*F* ^2^ > 2σ(*F* ^2^)] = 0.040
*wR*(*F* ^2^) = 0.092
*S* = 0.95
2833 reflections
219 parameters
H atoms treated by a mixture of independent and constrained refinement
Δρ_max_ = 0.11 e Å^−3^
Δρ_min_ = −0.17 e Å^−3^

### 

Data collection: *CrysAlis PRO* (Oxford Diffraction, 2010 ▶); cell refinement: *CrysAlis PRO*; data reduction: *CrysAlis PRO*; program(s) used to solve structure: *SIR92* (Altomare *et al.*, 1994 ▶); program(s) used to refine structure: *SHELXL97* (Sheldrick, 2008 ▶) within *WinGX* (Farrugia, 1999 ▶); molecular graphics: *PLATON* (Spek, 2009 ▶); software used to prepare material for publication: *PLATON*.

## Supplementary Material

Crystal structure: contains datablocks global, I. DOI: 10.1107/S1600536810050129/bt5426sup1.cif

Structure factors: contains datablocks I. DOI: 10.1107/S1600536810050129/bt5426Isup2.hkl

Additional supplementary materials:  crystallographic information; 3D view; checkCIF report

## Figures and Tables

**Table 1:** Hydrogen-bond geometry (Å, °)

*D*—H⋯*A*	*D*—H	H⋯*A*	*D*⋯*A*	*D*—H⋯*A*
N1*A*—H11*A*⋯O12^i^	1.00 (2)	1.71 (2)	2.688 (2)	164.2 (18)
N1*A*—H12*A*⋯O11	0.95 (2)	1.80 (2)	2.747 (2)	173.9 (17)
N41*A*—H41*A*⋯O52^ii^	0.83 (2)	2.39 (2)	3.216 (2)	170.8 (19)
N41*A*—H42*A*⋯O41*A*^iii^	0.99 (2)	1.91 (2)	2.873 (2)	164.8 (18)
O2—H2⋯O12	0.96 (2)	1.58 (2)	2.4897 (18)	156 (2)

Symmetry codes: (i) 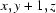; (ii) 
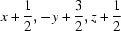
; (iii) 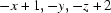.

**Fig. 1.**
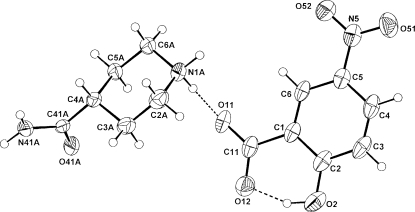
Molecular configuration and atom naming scheme for the INIPA cation and the 5-nitrosalicylate anion in (I). The inter-species hydrogen bond is shown as a dashed line and displacement ellipsoids are drawn at the 40% probability level.

**Fig. 2.**
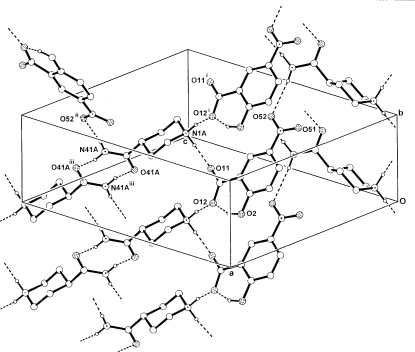
The hydrogen-bonded chain substructures in (I) showing the cyclic R22(8) amide–amide and cation–anion associations. Non-associative H atoms are omitted and hydrogen bonds are shown as dashed lines. For symmetry codes, see Table 1.

## supplementary crystallographic information

### Comment

The structures of a number of salts of the amide piperidine-4-carboxamide (isonipecotamide, INIPA) with a range of carboxylic acids, mainly aromatic, are now known (Smith & Wermuth, 2010*a*, 2010*b*, 2010*c*, Smith & Wermuth, 2010*d*,; Smith *et al.*, 2010). The title compound C_6_H_13_N_2_O^+^ C_7_H_4_NO_5_^-^ (I) was obtained from the 1:1 stoichiometric reaction of 5-nitrosalicylic acid with INIPA in methanol and the structure is reported here.

In (I) (Fig. 1) the cations and anions form hydrogn-bonded chain substructures through head-to-tail piperidinium *N*—*H*···O_carboxyl_ hydrogen bonds and through centrosymmetric cyclic head-to-head amide–amide hydrogen-bonding associations [graph set *R*^2^_2_(8) (Etter *et al.*, 1990)]. These chains are cross linked by amide *N*—*H*···O_carboxyl_ and piperidinium *N*—*H*···O_nitro_ associations to giving a two-dimensional sheet structure (Fig. 2). The amide-amide dimer association [the 'amide motif' (Allen *et al.*, 1998)] is relatively common among the INIPA salts (Smith & Wermuth, 2010*b*; Smith *et al.*, 2010).

The 5-nitrosalicylate anions are essentially planar [torsion angles for the carboxyl group (C2–C1–C11–O11), 178.30 (16)° and the nitro group (C4–C5–N5–O52), -175.57 (16)°], which is the usual conformation for this anion in its proton-transfer compounds (Smith *et al.*, 2005).

### Experimental

The title compound was synthesized by heating together under reflux for 10 minutes, 1 mmol quantities of piperidine-4-carboxamide (isonipecotamide) and 5-nitrosalicylic acid in 50 ml of methanol. After concentration to *ca* 30 ml, partial room temperature evaporation of the hot-filtered solution gave pale yellow prisms of the title compound from which a specimen was cleaved for the X-ray analysis.

### Refinement

Hydrogen atoms involved in hydrogen-bonding interactions were located by difference methods and their positional and isotropic displacement parameters were refined. Other H-atoms were included in the refinement at calculated positions using a riding-model approximation [C—H = 0.93–0.98 Å] and with *U*_iso_(H) = 1.2*U*_eq_(C).

### Crystal data

**Table:**

C_6_H_13_N_2_O^+^·C_7_H_4_NO_5_^−^	*F*(000) = 656
*M**_r_* = 311.30	*D*_x_ = 1.432 Mg m^−^^3^
Monoclinic, *P*2_1_/*n*	Melting point: 463 K
Hall symbol: -P 2yn	Mo *K*α radiation, λ = 0.71073 Å
*a* = 15.0442 (10) Å	Cell parameters from 3270 reflections
*b* = 5.5851 (3) Å	θ = 3.6–28.7°
*c* = 17.1939 (10) Å	µ = 0.12 mm^−^^1^
β = 91.466 (6)°	*T* = 200 K
*V* = 1444.22 (15) Å^3^	Prism, pale yellow
*Z* = 4	0.40 × 0.25 × 0.16 mm

### Data collection

**Table:**

Oxford Diffraction Gemini-S CCD-detector diffractometer	2833 independent reflections
Radiation source: Enhance (Mo)X-ray source	1850 reflections with *I* > 2σ(*I*)
graphite	*R*_int_ = 0.031
Detector resolution: 16.077 pixels mm^-1^	θ_max_ = 26.0°, θ_min_ = 3.6°
ω scans	*h* = −18→18
Absorption correction: multi-scan (*CrysAlis PRO*; Oxford Diffraction, 2010)	*k* = −6→6
*T*_min_ = 0.912, *T*_max_ = 0.980	*l* = −12→21
9191 measured reflections	

### Refinement

**Table:**

Refinement on *F*^2^	Primary atom site location: structure-invariant direct methods
Least-squares matrix: full	Secondary atom site location: difference Fourier map
*R*[*F*^2^ > 2σ(*F*^2^)] = 0.040	Hydrogen site location: inferred from neighbouring sites
*wR*(*F*^2^) = 0.092	H atoms treated by a mixture of independent and constrained refinement
*S* = 0.95	*w* = 1/[σ^2^(*F*_o_^2^) + (0.0486*P*)^2^] where *P* = (*F*_o_^2^ + 2*F*_c_^2^)/3
2833 reflections	(Δ/σ)_max_ < 0.001
219 parameters	Δρ_max_ = 0.11 e Å^−^^3^
0 restraints	Δρ_min_ = −0.17 e Å^−^^3^

### Special details

**Table:**

Geometry. Bond distances, angles *etc*. have been calculated using the rounded fractional coordinates. All su's are estimated from the variances of the (full) variance-covariance matrix. The cell e.s.d.'s are taken into account in the estimation of distances, angles and torsion angles
Refinement. Refinement of *F*^2^ against ALL reflections. The weighted *R*-factor *wR* and goodness of fit *S* are based on *F*^2^, conventional *R*-factors *R* are based on *F*, with *F* set to zero for negative *F*^2^. The threshold expression of *F*^2^ > σ(*F*^2^) is used only for calculating *R*-factors(gt) *etc*. and is not relevant to the choice of reflections for refinement. *R*-factors based on *F*^2^ are statistically about twice as large as those based on *F*, and *R*- factors based on ALL data will be even larger.

### Fractional atomic coordinates and isotropic or equivalent isotropic displacement parameters (Å^2^)

**Table:**

	*x*	*y*	*z*	*U*_iso_*/*U*_eq_	
O41A	0.44900 (9)	0.0250 (2)	0.90133 (7)	0.0575 (5)	
N1A	0.39663 (10)	0.5656 (3)	0.69549 (9)	0.0452 (6)	
N41A	0.54984 (11)	0.2658 (4)	0.96084 (10)	0.0480 (6)	
C2A	0.49383 (12)	0.5253 (3)	0.70538 (12)	0.0545 (7)	
C3A	0.51390 (12)	0.3391 (3)	0.76686 (10)	0.0516 (7)	
C4A	0.47324 (11)	0.4101 (3)	0.84420 (9)	0.0423 (6)	
C5A	0.37359 (11)	0.4529 (3)	0.83207 (10)	0.0451 (6)	
C6A	0.35478 (11)	0.6369 (3)	0.76956 (10)	0.0463 (6)	
C41A	0.48930 (12)	0.2177 (3)	0.90488 (10)	0.0435 (6)	
O2	0.40541 (10)	−0.1530 (2)	0.42604 (8)	0.0605 (5)	
O11	0.32739 (8)	0.1529 (2)	0.63160 (7)	0.0507 (4)	
O12	0.39253 (9)	−0.1469 (3)	0.57010 (7)	0.0600 (5)	
O51	0.16400 (10)	0.7176 (3)	0.34253 (8)	0.0729 (6)	
O52	0.17024 (9)	0.7584 (2)	0.46713 (8)	0.0562 (5)	
N5	0.19077 (10)	0.6528 (3)	0.40756 (9)	0.0510 (6)	
C1	0.32654 (11)	0.1565 (3)	0.49283 (9)	0.0388 (6)	
C2	0.35489 (12)	0.0445 (3)	0.42422 (10)	0.0460 (6)	
C3	0.32936 (13)	0.1368 (4)	0.35198 (10)	0.0550 (7)	
C4	0.27649 (13)	0.3354 (4)	0.34625 (10)	0.0529 (7)	
C5	0.24816 (11)	0.4452 (3)	0.41392 (9)	0.0423 (6)	
C6	0.27300 (11)	0.3580 (3)	0.48665 (9)	0.0396 (6)	
C11	0.35061 (12)	0.0510 (3)	0.57113 (10)	0.0443 (6)	
H4A	0.50110	0.55900	0.86250	0.0510*	
H11A	0.3874 (12)	0.689 (4)	0.6540 (12)	0.065 (6)*	
H12A	0.3699 (12)	0.422 (4)	0.6763 (11)	0.063 (6)*	
H21A	0.52260	0.67460	0.72000	0.0650*	
H22A	0.51770	0.47330	0.65630	0.0650*	
H31A	0.57780	0.32230	0.77390	0.0620*	
H32A	0.49000	0.18580	0.75010	0.0620*	
H41A	0.5753 (13)	0.398 (4)	0.9620 (11)	0.054 (7)*	
H42A	0.5568 (13)	0.147 (4)	1.0029 (12)	0.075 (7)*	
H51A	0.34480	0.30340	0.81770	0.0540*	
H52A	0.34870	0.50710	0.88050	0.0540*	
H61A	0.29100	0.65250	0.76110	0.0560*	
H62A	0.37790	0.79110	0.78630	0.0560*	
H2	0.4119 (15)	−0.184 (4)	0.4809 (14)	0.109 (9)*	
H3	0.34850	0.06260	0.30700	0.0660*	
H4	0.25970	0.39640	0.29770	0.0630*	
H6	0.25380	0.43450	0.53130	0.0470*	

### Atomic displacement parameters (Å^2^)

**Table:**

	*U*^11^	*U*^22^	*U*^33^	*U*^12^	*U*^13^	*U*^23^
O41A	0.0736 (9)	0.0473 (8)	0.0500 (8)	0.0056 (7)	−0.0280 (7)	−0.0127 (6)
N1A	0.0518 (10)	0.0467 (10)	0.0368 (9)	−0.0015 (8)	−0.0070 (7)	−0.0078 (8)
N41A	0.0472 (10)	0.0463 (10)	0.0495 (10)	0.0124 (9)	−0.0208 (8)	−0.0123 (9)
C2A	0.0482 (12)	0.0624 (13)	0.0531 (12)	0.0009 (10)	0.0056 (9)	−0.0120 (10)
C3A	0.0439 (11)	0.0576 (12)	0.0530 (12)	0.0122 (9)	−0.0014 (9)	−0.0134 (10)
C4A	0.0421 (10)	0.0420 (10)	0.0420 (10)	0.0090 (8)	−0.0125 (8)	−0.0149 (8)
C5A	0.0422 (10)	0.0580 (11)	0.0348 (10)	0.0155 (9)	−0.0066 (8)	−0.0066 (9)
C6A	0.0429 (10)	0.0561 (11)	0.0396 (10)	0.0127 (9)	−0.0066 (8)	−0.0080 (9)
C41A	0.0418 (10)	0.0454 (11)	0.0426 (10)	0.0189 (9)	−0.0139 (8)	−0.0195 (9)
O2	0.0834 (10)	0.0489 (8)	0.0501 (8)	−0.0077 (8)	0.0216 (8)	−0.0104 (7)
O11	0.0597 (8)	0.0621 (8)	0.0303 (7)	−0.0163 (7)	−0.0002 (6)	−0.0097 (6)
O12	0.0762 (10)	0.0563 (8)	0.0474 (8)	0.0017 (8)	0.0024 (7)	−0.0007 (7)
O51	0.0929 (11)	0.0800 (11)	0.0455 (8)	−0.0009 (8)	−0.0025 (8)	0.0174 (7)
O52	0.0630 (9)	0.0544 (8)	0.0516 (9)	−0.0081 (7)	0.0075 (7)	−0.0025 (7)
N5	0.0580 (10)	0.0546 (10)	0.0406 (10)	−0.0209 (9)	0.0067 (8)	0.0033 (8)
C1	0.0423 (10)	0.0414 (10)	0.0331 (10)	−0.0211 (9)	0.0064 (7)	−0.0067 (8)
C2	0.0590 (12)	0.0407 (10)	0.0390 (11)	−0.0221 (9)	0.0148 (9)	−0.0066 (9)
C3	0.0810 (15)	0.0528 (12)	0.0322 (11)	−0.0223 (11)	0.0224 (10)	−0.0069 (9)
C4	0.0715 (14)	0.0585 (12)	0.0291 (10)	−0.0264 (11)	0.0099 (9)	0.0020 (9)
C5	0.0483 (11)	0.0438 (11)	0.0352 (10)	−0.0184 (9)	0.0073 (8)	0.0017 (8)
C6	0.0430 (10)	0.0457 (10)	0.0302 (9)	−0.0230 (9)	0.0056 (7)	−0.0079 (8)
C11	0.0453 (11)	0.0505 (11)	0.0369 (11)	−0.0208 (9)	0.0001 (8)	−0.0070 (9)

### Geometric parameters (Å, °)

**Table:**

O41A—C41A	1.236 (2)	C2A—H22A	0.9700
O2—C2	1.340 (2)	C2A—H21A	0.9700
O11—C11	1.243 (2)	C3A—H31A	0.9700
O12—C11	1.273 (2)	C3A—H32A	0.9700
O51—N5	1.233 (2)	C4A—H4A	0.9800
O52—N5	1.228 (2)	C5A—H51A	0.9700
O2—H2	0.96 (2)	C5A—H52A	0.9700
N1A—C6A	1.489 (2)	C6A—H62A	0.9700
N1A—C2A	1.485 (2)	C6A—H61A	0.9700
N41A—C41A	1.335 (2)	C1—C6	1.386 (2)
N1A—H12A	0.95 (2)	C1—C11	1.505 (2)
N1A—H11A	1.00 (2)	C1—C2	1.411 (2)
N41A—H41A	0.83 (2)	C2—C3	1.390 (3)
N41A—H42A	0.99 (2)	C3—C4	1.367 (3)
N5—C5	1.448 (2)	C4—C5	1.392 (2)
C2A—C3A	1.508 (3)	C5—C6	1.385 (2)
C3A—C4A	1.530 (2)	C3—H3	0.9300
C4A—C5A	1.527 (2)	C4—H4	0.9300
C4A—C41A	1.513 (2)	C6—H6	0.9300
C5A—C6A	1.509 (2)		
			
C2—O2—H2	102.4 (13)	C5A—C4A—H4A	109.00
C2A—N1A—C6A	112.29 (14)	C3A—C4A—H4A	109.00
C2A—N1A—H12A	108.5 (12)	C4A—C5A—H52A	109.00
C6A—N1A—H11A	111.8 (12)	C6A—C5A—H51A	109.00
H11A—N1A—H12A	106.5 (17)	C4A—C5A—H51A	109.00
C6A—N1A—H12A	109.7 (11)	C6A—C5A—H52A	109.00
C2A—N1A—H11A	107.8 (11)	H51A—C5A—H52A	108.00
H41A—N41A—H42A	122.7 (18)	H61A—C6A—H62A	108.00
C41A—N41A—H42A	116.8 (12)	C5A—C6A—H61A	110.00
C41A—N41A—H41A	120.1 (13)	C5A—C6A—H62A	110.00
O51—N5—O52	122.14 (16)	N1A—C6A—H61A	109.00
O51—N5—C5	118.93 (15)	N1A—C6A—H62A	110.00
O52—N5—C5	118.93 (14)	C2—C1—C11	120.22 (15)
N1A—C2A—C3A	111.33 (15)	C6—C1—C11	120.82 (14)
C2A—C3A—C4A	110.68 (14)	C2—C1—C6	118.90 (15)
C3A—C4A—C41A	110.79 (14)	O2—C2—C1	121.94 (15)
C5A—C4A—C41A	110.14 (14)	O2—C2—C3	118.03 (16)
C3A—C4A—C5A	109.56 (13)	C1—C2—C3	120.02 (16)
C4A—C5A—C6A	111.69 (14)	C2—C3—C4	120.82 (17)
N1A—C6A—C5A	110.57 (14)	C3—C4—C5	119.17 (16)
O41A—C41A—N41A	122.38 (17)	N5—C5—C6	119.77 (14)
N41A—C41A—C4A	116.62 (16)	C4—C5—C6	121.25 (16)
O41A—C41A—C4A	120.99 (15)	N5—C5—C4	118.98 (15)
N1A—C2A—H21A	109.00	C1—C6—C5	119.84 (15)
N1A—C2A—H22A	109.00	O11—C11—C1	120.16 (15)
C3A—C2A—H21A	109.00	O12—C11—C1	115.81 (15)
C3A—C2A—H22A	109.00	O11—C11—O12	123.99 (16)
H21A—C2A—H22A	108.00	C2—C3—H3	120.00
C2A—C3A—H32A	110.00	C4—C3—H3	120.00
C4A—C3A—H31A	109.00	C3—C4—H4	120.00
C2A—C3A—H31A	110.00	C5—C4—H4	120.00
C4A—C3A—H32A	109.00	C1—C6—H6	120.00
H31A—C3A—H32A	108.00	C5—C6—H6	120.00
C41A—C4A—H4A	109.00		
			
C6A—N1A—C2A—C3A	56.83 (19)	C6—C1—C2—C3	0.2 (3)
C2A—N1A—C6A—C5A	−56.01 (18)	C11—C1—C2—O2	−1.8 (3)
O51—N5—C5—C4	4.5 (2)	C11—C1—C2—C3	177.28 (17)
O51—N5—C5—C6	−174.74 (16)	C2—C1—C6—C5	0.2 (2)
O52—N5—C5—C4	−175.57 (16)	C11—C1—C6—C5	−176.88 (16)
O52—N5—C5—C6	5.2 (2)	C2—C1—C11—O11	178.30 (16)
N1A—C2A—C3A—C4A	−56.37 (19)	C2—C1—C11—O12	−3.8 (2)
C2A—C3A—C4A—C5A	55.43 (18)	C6—C1—C11—O11	−4.7 (3)
C2A—C3A—C4A—C41A	177.15 (14)	C6—C1—C11—O12	173.26 (16)
C5A—C4A—C41A—O41A	49.2 (2)	O2—C2—C3—C4	178.81 (18)
C5A—C4A—C41A—N41A	−132.02 (17)	C1—C2—C3—C4	−0.3 (3)
C3A—C4A—C41A—O41A	−72.2 (2)	C2—C3—C4—C5	0.0 (3)
C3A—C4A—C5A—C6A	−55.62 (18)	C3—C4—C5—N5	−178.83 (17)
C41A—C4A—C5A—C6A	−177.72 (14)	C3—C4—C5—C6	0.4 (3)
C3A—C4A—C41A—N41A	106.61 (18)	N5—C5—C6—C1	178.76 (15)
C4A—C5A—C6A—N1A	55.72 (18)	C4—C5—C6—C1	−0.5 (3)
C6—C1—C2—O2	−178.84 (16)		

### Hydrogen-bond geometry (Å, °)

**Table:**

*D*—H···*A*	*D*—H	H···*A*	*D*···*A*	*D*—H···*A*
N1A—H11A···O12^i^	1.00 (2)	1.71 (2)	2.688 (2)	164.2 (18)
N1A—H12A···O11	0.95 (2)	1.80 (2)	2.747 (2)	173.9 (17)
N41A—H41A···O52^ii^	0.83 (2)	2.39 (2)	3.216 (2)	170.8 (19)
N41A—H42A···O41A^iii^	0.99 (2)	1.91 (2)	2.873 (2)	164.8 (18)
O2—H2···O12	0.96 (2)	1.58 (2)	2.4897 (18)	156 (2)
C2A—H22A···O2^iv^	0.97	2.57	3.450 (2)	150
C6A—H61A···O11^v^	0.97	2.60	3.263 (2)	126
C6A—H62A···O41A^i^	0.97	2.58	3.417 (2)	145

Symmetry codes: (i) *x*, *y*+1, *z*; (ii) *x*+1/2, −*y*+3/2, *z*+1/2; (iii) −*x*+1, −*y*, −*z*+2; (iv) −*x*+1, −*y*, −*z*+1; (v) −*x*+1/2, *y*+1/2, −*z*+3/2.
